# Self-reported sex differences in high-functioning adults with autism: a meta-analysis

**DOI:** 10.1186/s13229-018-0216-6

**Published:** 2018-05-18

**Authors:** R. L. Moseley, R. Hitchiner, J. A. Kirkby

**Affiliations:** Department of Psychology, Bournemouth University, Fern Barrow, Poole, Dorset, BH12 5BB UK

**Keywords:** Sex, Gender, Self-report, RAADS-R

## Abstract

**Background:**

Sex differences in autistic symptomatology are believed to contribute to the mis- and missed diagnosis of many girls and women with an autism spectrum condition (ASC). Whilst recent years have seen the emergence of clinical and empirical reports delineating the profile of young autistic girls, recognition of sex differences in symptomatology in adulthood is far more limited.

**Methods:**

We chose here to focus on symptomatology as reported using a screening instrument, the Ritvo Autism Asperger Diagnostic Scale-Revised (RAADS-R). In a meta-analysis, we pooled and analysed RAADS-R data from a number of experimental groups. Analysis of variance (ANOVA) searched for the presence of main effects of Sex and Diagnosis and for interactions between these factors in our sample of autistic and non-autistic adults.

**Results:**

In social relatedness and circumscribed interests, main effects of Diagnosis revealed that as expected, autistic adults reported significantly greater lifetime prevalence of symptoms in these domains; an effect of Sex, in circumscribed interests, also suggested that males generally reported more prevalent symptoms than females. An interaction of Sex and Diagnosis in language symptomatology revealed that a normative sex difference in language difficulties was attenuated in autism. An interaction of Sex and Diagnosis in the sensorimotor domain revealed the opposite picture: a lack of sex differences between typically developing men and women and a greater prevalence of sensorimotor symptoms in autistic women than autistic men.

**Conclusions:**

We discuss the literature on childhood sex differences in relation to those which emerged in our adult sample. Where childhood sex differences fail to persist in adulthood, several interpretations exist, and we discuss, for example, an inherent sampling bias that may mean that only autistic women most similar to the male presentation are diagnosed. The finding that sensorimotor symptomatology is more highly reported by autistic women is a finding requiring objective confirmation, given its potential importance in diagnosis.

**Electronic supplementary material:**

The online version of this article (10.1186/s13229-018-0216-6) contains supplementary material, which is available to authorized users.

## Background

Females with autism are historically underdiagnosed. In cognitively impaired children, autism diagnosis is currently estimated at two boys to every girl in cognitively impaired children, whereas in those who are higher-functioning, estimates range from 5.7, 11 or 15.7 boys to every girl (see [1, 2]). Most recently within the UK (Scotland, specifically), diagnostic ratios were put at 3.5 males to every female in autistic children and adolescents, and two males to every female in adults [3]. A recent review of diagnosis internationally came to a similar diagnostic ratio, in children, of three boys diagnosed to every girl [4]. Convincing arguments from genetic research, beyond the scope of the present article, suggest that the prevalence of autism could be genuinely lower in females [2, 5–8], but in so far as those who are diagnosed, diagnostic rates appear to reflect a kind of ‘bimodal distribution’, with the more severely impaired autistic females likely to be detected in childhood and those without intellectual disability and with subtler presentations likely to be either missed or diagnosed later in life [1, 9]. The fact that age of diagnosis is on average later in autistic females than males corroborates the known difficulty identifying girls and women and corroborates the calls from the autistic and the scientific community for research into the female autistic phenotype [5, 10].

Clinical reports and empirical studies continue to crystallise the female phenotype as it appears in young girls, though it must be noted that differences in sampling techniques and methodologies make comparison of findings somewhat opaque. Several studies of early childhood suggest that differences may become more apparent with age, finding no significant differences between male and female infants and toddlers in autistic symptomatology within broad domains [11–13]. A more detailed look at each symptom category, as children age, reveals the emergence of considerable differences. With consideration of the core diagnostic impairments in social communication and interaction [14], girls with autism are believed to be equivalent to their male peers in core difficulties with social *understanding* [5, 15]; reports that autistic girls exhibit greater social impairment [16–18] may be subject to the fact that less severe presentations of autism (i.e. high-functioning autism) are less likely to be recognised and thus diagnosed in girls [19–22]). The *expressive* behaviours of girls with autism, such as in making reciprocal conversation and displaying appropriate non-verbal behaviour and gestures, do however tend to outpass male peers [5, 23]; this is starkly illustrated by Hiller et al. [24], who found that whilst girls are more likely to use social gestures, their usage does not reflect underlying understanding. The fact that gestures may be unusually ‘vivid’, characterised by increased energy [23], could potentially say something of their learned nature. Young girls with autism are also known to be far more likely than boys to engage in complex imitation [24], which is problematic given the central featuring of imitative abilities in gold-standard diagnostic tests.

Autistic girls are more likely to correspond to Wing and Gould’s [25] ‘active but odd’ category and tend away from ‘autistic aloneness’ [26, 27]. Indeed, where males with autism may withdraw from the more active games of their peers [24, 28], autistic girls are believed to be more socially motivated [29, 30] and, like non-autistic girls, to spend time chatting with friends as opposed to engaging in activities like sports or gaming [30–32]. Whilst these studies highlight similarities between autistic and non-autistic girls in female friendships, they do note that autistic girls struggle with managing conflict in relationships, and that social time is exhausting to them. This may be because, unlike autistic boys, autistic girls appear especially adept at skilfully managing social interaction through mimicking and rote-learnt strategies [24, 33, 34]. Qualitative investigation of these strategies suggest masterful adaptation where girls describe empathetic approaches as piggybacking on excellent memory and adherence to a learnt “social code” via observation and subsequent imitation [35]. Quantitative attempts to capture these abilities show a discrepancy between the scores of women on mentalising tests and core autistic traits (measuring internal disposition and core ability) and their outwards sociocommunicative performance in the Autism Diagnostic Observation Schedule (ADOS-G) [36]. The masking skills of girls and women can unfortunately confound diagnosis, as does a lack of awareness of the female autistic phenotype in professionals and the gender stereotypes which cast socially impaired girls as ‘shy’ and socially impaired boys as ‘unresponsive’ [37]. Less disruptive, with fewer externalising and more internalising problems at school age [38–41], autistic girls are more likely to be mothered or accepted by non-autistic girls as fringe members of female social groups at least until adolescence, when female friendships require considerable social adroitness [35].

Autistic girls are also less likely than boys to stand out in the diagnostic domain of restricted and repetitive interests and behaviour, where they tend to exhibit fewer classically autistic symptoms like lining objects up and fascination with small parts [15, 17, 42–46]. Indeed, fascination with small parts and mechanical objects, in early-diagnosed children, is predictive of their being male [24]. Special interests, in girls, tend to be less eccentric and more age- and gender-appropriate (for example ponies or boybands), collecting things like stickers or shells, or obsessional behaviour with toys [24] but equal to those of autistic boys (and different from non-autistic girls) in their intensity [5, 33]. Autistic girls are more likely to engage in pretend play than autistic boys and may appear to have rich inner lives which under closer scrutiny may be seen to be extraordinarily scripted and repetitive [33, 39, 40, 47]. Sensory processing differences, which also fall within this diagnostic domain, are apparently equally apparent [33, 48], but research in this domain is limited; others report greater abnormalities in touch, taste and smell in autistic girls [49].

With the literature focused on childhood presentation, autistic adults are a neglected population in research and less is known about whether these sex differences in autistic symptomatology persist. Both autistic men and women differ from *non-autistic adults* in the attention they pay to faces [50], though interestingly this study did not replicate the trend seen in autistic males to fixate more on the mouth area [51, 52]. These three studies found abnormalities in social attention (as reflected by eye-gaze) to correlate with social competence, emotion recognition and autistic symptomatology respectively [50–52], so it is therefore perhaps unsurprising that difficulties with emotion recognition remain equally prevalent and equivalent in autistic men and women [1, 53], and likewise no differences were seen in empathy and systemizing, the drive to fit the world into rule-based systems [53, 54]. This last finding is particularly note-worthy given that normative sex differences in these domains appear to be attenuated in men and women with autism, a finding corroborated by a large-scale survey that revealed that men and women with autism are more similar to each other than are typically developing men and women [55].

Notwithstanding these similarities, other reports suggest that autism continues to present differently in males and females once they reach adulthood. Lai et al. [53] observed lower scores for women on the sociocommunicative aspects of the ADOS-G [56], seemingly consistent with the expressive skills of autistic females mentioned above, and reports of more sensory issues. There have been some reports of advantages for autistic women over autistic men in executive function and processing speed [1, 54, 57], which may partially explain their success in camouflage [34, 35, 58, 59]. Not all studies, however, have found differences between autistic men and women in executive function (for example, a lack of difference in response inhibition [54]): it is important to note that ‘executive function’, as a construct, in fact consists of multiple processes, each with distinct developmental trajectories, which are difficult to tease apart and to test in an ecologically valid way, hence the inconsistencies across autism research [60]. Indeed, some reports of sex differences in executive function in autistic children have reported patterns in the reverse, with poorer response inhibition and greater perseveration in autistic girls than boys [61, 62].

Outside of comparative tests in the laboratory, few studies have compared the real life outcomes in autistic men and women. A qualitative analysis by Baldwin and Costley [63] suggested that women might also have greater success than men in being able to study in higher education, though they also self-reported higher rates of mental illness. The same study suggested some interesting reversals of childhood trends: women were more likely than men to highlight difficulties with social interaction as the worst aspects of their employment history, were less likely to aspire to marriage or romantic relationships and more likely to prefer their own company, in contrast to the apparently higher social motivation seen in childhood. Whilst this study featured an impressive sample size, quantitative validation of these tantalising hints would be important.

A common theme throughout the limited literature in adults concerns the struggles that autistic women face in obtaining a diagnosis [1, 58, 59, 63]. The implications of this difficulty are potentially immense, such that many individuals lack support and treatment for their symptomatology [22, 64]. For this reason, the present study aimed to further the limited literature on the symptomatic differences between autistic men and women. We chose to do so utilising an established screening test which is employed in local diagnostic services in South-West England: the Ritvo Autism Asperger Diagnostic Scale-Revised (RAADS-R [65]). Sample size is always immensely problematic in comparing males and females with autism, given the diagnostic bottleneck which results in many more males than females being identified [22]. For this reason, we sought to supplement our own data by pooling it with that from participating researchers who had also used the RAADS-R. We thus examined self-report ratings made by autistic and non-autistic adults of symptomatology in four domains: social relatedness, circumscribed interests, language and sensorimotor abnormalities.

## Methods

We adopted the two-factorial design recommended by Lai et al. [5]: by comparing autistic men and women to each other as well as to typically developing men and women, it is possible to tease out normative sex differences in cognition which may or may not be present in autism. The focus for comparison was scores in the RAADS-R domains of social relatedness, circumscribed interests, sensory motor (henceforth sensorimotor) and language symptoms.

To supplement data gathered by our research group, we conducted a meta-analysis of studies which had used the RAADS-R. Below we describe our final selection of participants and the process of our meta-analysis, but details of the participant cohorts involved are given fully in Additional file 1. Ethical approval for the study was given by Bournemouth University Ethics Committee.

### Participants

We obtained a total 961 datasets: 179 typically developing (TD) men, 528 typically developing women, 118 autistic men, and 136 autistic women (see Additional file 1) (Table 1). To ensure data quality, some control participants who scored particularly highly on the RAADS-R (and so might potentially be undiagnosed autistics) were removed (see Additional file 1, for details), leaving 137 TD men, 464 TD women, 118 autistic men, and 136 autistic women. In attempts to objectively create more evenly sized and age-matched groups, we used freely available software [66] to reduce the number of TD females by selecting those best matched in age to the other groups. Therefore, the final participants included in our analysis were 137 TD men, 136 TD women, 118 autistic men, and 136 autistic women. Whilst significant age differences remained between all four groups (*F* [3, 523] = 3.230, *p* = .022), no significant age differences remained in three of the four contrasts of interest for this analysis: namely, between TD men and men with autism spectrum conditions (ASC) (*p* = .192), TD females and females with ASC (*p* = .944), and between autistic men and women (*p* = .194). TD women included in the study were significantly older than TD men (*t* [271] = 2.635, *p* = .009).

**Table 1:** Average age in years (standard deviation in brackets) for each experimental group. The number of participants included from each source is displayed to the right

	Age (years)	Source
Typically developing men (*n* = 137)	33.1 (13.5)	Bournemouth University (*n* = 30) Kirkovski/Fitzgerald group (*n* = 12) Libero/Kana group (*n* = 13) Schwartzman/Kapp group (*n* = 82)
Typically developing women (*n* = 136)	37.4 (13.8)	Bournemouth University (*n* = 16) Kirkovski/Fitzgerald group (*n* = 4) Schwartzman/Kapp group (*n* = 116)
Autistic men (*n* = 118)	35.3 (13.4)	Bournemouth University (*n* = 34) Kirkovski/Fitzgerald group (n = 13) Libero/Kana group (*n* = 5) Schwartzman/Kapp group (*n* = 84)
Autistic women (*n* = 136)	37.5 (14)	Bournemouth University (*n* = 35) Kirkovski/Fitzgerald group (*n* = 12) Libero/Kana group (*n* = 14) Schwartzman/Kapp group (*n* = 57)

As we were unable to obtain details of IQ, we were unable to match participants in this variable; however, all individuals are assumed to be of average or above-average IQ due to the nature of the recruitment process and the studies they participated in (see Additional file 1). We do not possess details of comorbid psychiatric disorders or use of psychotropic medication for all datasets, and so cannot confirm that all participants were medication-free or without additional psychiatric conditions.

### Materials

The Ritvo Autism Asperger Diagnostic Scale-Revised (RAADS-R [65]) is an 80-item self-report questionnaire recommended by the National Institute of Health and Care Excellence [67] in Great Britain to screen adults of average to above-average intelligence for an autism spectrum condition. Although it has been used in research as a self-report measure, the RAADS-R was designed to be completed in clinical settings with the assistance of a clinician. ‘Diagnostic Scale’ is somewhat misleading [68]: this test functions rather as a screening instrument or, as the authors intended, as just one part of a comprehensive assessment rather than a stand-alone diagnostic instrument. The revised version of the original scale was standardised on 201 autistic individuals (145 males) and 578 non-autistic TD (248 males), collected in nine centres on three continents, and like the original is based on diagnostic criteria for autism and Asperger syndrome in DSM-IV-TR and ICD-10 (criteria that were retained in DSM-V). In this large study, the test showed high specificity in its ability to distinguish between TD and autistic individuals whose diagnoses had been independently confirmed (no false positives). Only six of 201 autistic participants scored below 65 and were consequently unidentified (97% sensitivity). The test also showed good test-retest reliability and high concurrent validity (95.59%) with other popular tests for ASC such as the Social Responsiveness Scale Adult Research version [69]. It has been validated for use in other languages [70] and shortened to a 14-item version with demonstrated capacity to discriminate between ASC and some commonly comorbid psychiatric conditions [71].

The RAADS-R yields four subscales based on symptom areas from DSM-IV-TR [72] and ICD-10 [73], which themselves have high internal consistency. These domains are social relatedness (e.g. ‘I often don’t know how to act in social situations’), circumscribed interests (e.g. ‘I only like to talk to people who share my special interests’), sensorimotor (e.g. ‘I always notice how food feels in my mouth. This is more important to me than how it tastes’) and language (e.g. ‘I have a hard time figuring out what some phrases mean, like “you are the apple of my eye”’). Each item is scored in order of its emergence and current occurrence, with ‘True now and when I was young’ scored at 3; ‘True only now’ scored at 2; ‘True only when I was younger than 16’ scored at 1; and ‘Never true’ scored at 0. (This scoring is reversed for negative items, such as ‘I can put myself in other people’s shoes’).

### Procedure

In order to obtain a sizeable sample, we supplemented our data with that collected by other authors in a meta-analysis [74]. Inclusion criteria were that (1) studies must include clinically diagnosed autistic and non-autistic participants, on whom the RAADS-R had been conducted; (2) participants must be adults (that is, aged 18 or above); and (3) only studies using the RAADS-R, not the original Ritvo Autism Asperger Diagnostic Scale or the newer 14-item version [71] would be included. This therefore stipulated criteria 4), that only studies occurring between 2011 (the publication of the RAADS-R) and the present year of 2017 would be included. Exclusion criteria included (1) studies involving other clinical but non-autistic populations which were being screened for autistic traits (e.g. [65, 75]); (2) studies which used the RAADS-R to assume the presence of autism but did not confirm the diagnosis with participants (e.g. [76]); (3) studies written in languages other than English; and 4) or reviews citing the RAADS-R which did not include actual data.

We searched three online databases (Web of Science, PubMed, Science Direct) with the search command: ‘Ritvo Autism Asperger Diagnostic Scale-Revised’. We also used Google Scholar to identify all publications which had cited Ritvo et al.’s publication of their scale. With some overlap and much redundancy, we obtained 6 search results from Web of Science, 4 from PubMed, 87 from Science Direct, and 85 from Google Scholar (see Additional file 1). Sorting through these citations with our criteria in mind, we identified 16 relevant studies and contacted 8 research groups (see Additional file 1). We received useable datasets from three of these (see Additional file 1). We ensured the data was numerically coded in the same way as our own (one for female, two for male, for example) before collating it in SPSS (Statistical Programme for the Social Sciences).

Statistical analysis examined scores on the social relatedness, circumscribed interests, language and sensorimotor subscales of the RAADS-R. For each domain, separate two-way ANOVAs included between-subjects factors of Diagnosis (autistic vs. TD) and Sex (female vs. male). Interactions between Diagnosis and Sex, in this context, indicate that sex differences are attenuated or increased by the presence or lack of an autism diagnosis. The presence of an interaction thus motivated post hoc comparisons between males and females within the TD and within the autistic group.

## Results

**Fig. 1:**
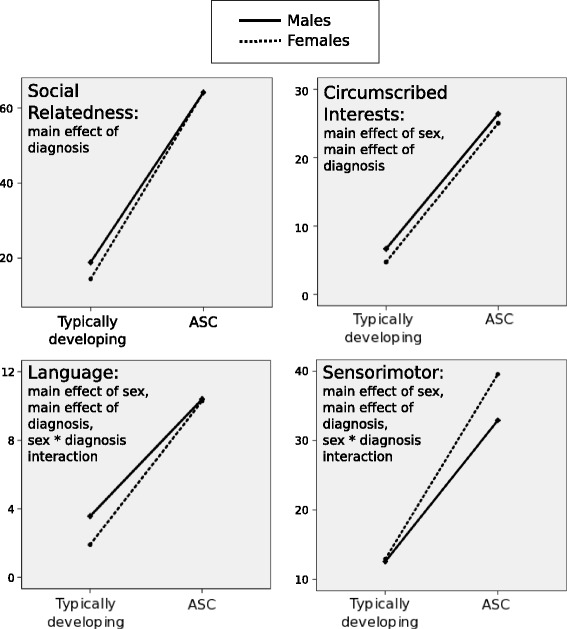
Average scores for each group in each RAADS-R domain. Average scores, reflecting self-reported symptomatology, for typically developing men, typically developing women, autistic men and autistic women in each domain of the RAADS-R

Effects of sex and diagnosis were examined for each RAADS-R domain independently and averages for each group can be seen in Fig. 1. In the social relatedness domain, typically developed men scored an average of 18.9 (SD 10.8), typically developing women an average of 14.5 (SD 10.9), autistic men an average of 64.2 (SD 23) and autistic women an average of 64.2 (SD 19.7). A main effect of Diagnosis (*F* [1, 523] = 1068.299, *p* = .000) reflected that autistic participants reported significantly higher prevalence of social problems than typically developed individuals—a finding corroborating the original paper [65] and subsequent validations of the test [70, 71]. In the same vein, examination of the circumscribed interests domain revealed a main effect of Diagnosis (*F* [1, 523] = 904.268, *p* < .001), with typically developing men and women reporting lower symptoms on average (men 6.7 [SD 4.9], women 4.7 [SD 4.1]) than autistic men and women (men 26.4 [SD 10.1], women 25 [SD 9.8]). There was also a main effect of Sex (*F* [1, 523 = .6.080, *p* = .014) reflecting that males generally report more behaviours than women in the circumscribed interests domain.

In the language domain, typically developing men scored on average 3.6 (SD 2.5); women an average 1.9 [SD 1.8]); autistic men an average 10.4 [SD 4.6]; and autistic women an average 10.3 [SD 5.2]. A main effect of Sex (*F* [1, 523] = 7.333, *p* = .007) and a main effect of Diagnosis (*F* [1, 523] = 542.630, *p* < .001) reflected that women generally reported lower scores in autistic language symptomatology than men and that, as expected, individuals with autism reported significantly more symptoms than TD controls. A significant interaction between Sex and Diagnosis (*F* [1, 523, *p* = 5.707, *p* = .017) motivated post hoc tests, which revealed that scores differed significantly between typically developing men and women (*t* [271] = 6.311, *p* < .001) but not between autistic men and women (*p* = .866).

Highest scores in the sensorimotor domain were seen in autistic women (average 39.6 [SD 12.6]), followed by autistic men (average 32.9 [SD 11.6]), typically developing women (average 12.9 [SD 7.6]) and typically developing men (average 12.5 [SD 7.2]). A significant main effect of Sex (*F* [1, 523] = 16.235, *p* < .001) reflected lower self-reported sensorimotor abnormalities in women, and a main effect of Diagnosis (*F* [1, 523] = 726.807, *p* < .001) reflected greater symptomatology in the autistic group. An interaction between Sex and Diagnosis (*F* [1, 523, *p* = .12.983, *p* < .001), in this domain, reflected a surprising lack of difference between TD men and women (*p* = .252) and a significant difference between autistic men and women (*t* [252] = 4.346, *p* < .001).

## Discussion

For the purpose of early identification, prior investigations of sex differences in autism have predominantly focused on child samples. Given the known difficulty identifying females with autism and the aptitude of many female and *male* individuals to camouflage their symptoms [32, 34, 35, 58, 59, 63], a substantial unidentified population reach adulthood before being diagnosed [1, 64]. We consequently aimed to extend the small literature on how autistic symptomatology presents in autistic men and women through investigating a commonly used self-report measure, the Ritvo Autism Asperger Diagnostic Scale Revised (RAADS-R [65]). Studying sex differences as and if they emerge in screening instruments may be particularly important if these are considered frontline measures used in triage, as the RAADS-R happens to be in our area. To increase the power of our analysis, we conducted a meta-analysis, gathering data from several research groups. We discuss first the areas in which autistic men and women presented similarly and then the domain in which they differed.

Whilst the now extensive literature on sex differences in autistic children emphasises the divergence between girls and boys, previous investigations in autistic adults have reported similar competence in emotion recognition [1, 53] and even the *attenuation* of normative sex differences in empathising and systemising [53–55]. These findings align with a theoretical perspective that links ASC with the masculinization of brain and behaviour [77–80]. In the domain of social relatedness, we did not find that normative sex differences were attenuated in autism but that autistic men and women were alike in their quantification of symptomology. This is consistent with childhood impairments in the social domain, which appear to be of equal severity in boys and girls [15]. Previous studies have, however, noted a stark divergence between core social understanding and outward expressive social interaction in females [24], which indeed appears to be somewhat more typical due to skilled social mimicry [5, 23, 24, 33]. Our data appears to reflect the shared core disability in social understanding, as the social difficulties of autistic women were reported as no less prominent than those of autistic men. A qualitative difference previously reported relating to autistic women’s heightened concern over social interaction [63] was not here quantified in reports of greater prevalence of social problems. Of note, however, is the self-report nature of our data: this greater concern over social competence could possibly have served to hide the better *expressive* social skills evinced in previous studies, if autistic women are inaccurate reporters. As in other studies of sex differences in autistic adults [53], the validity of our self-report measures depends on the self-reflective capacity of participants, differences, or in this case *lack* of sex differences, thus require independent, objective ratification.

This point holds true when we consider a lack of sex differences between autistic men and women in the circumscribed interests domain, despite a main effect of sex reflecting a general tendency for men to report more symptomatology in this domain. The ‘circumscribed interests’ domain of the RAADS-R aligns itself with the repetitive and restricted behaviours and interest (RRBI) diagnostic criterion [14], including items describing fixated and unusual interests which dominate conversation, detail-level focusing, adherence to fixed routines and difficulties with change, and enjoyment of lists and categorisation. It differs, however, from RRBI as conceptualised in diagnostic instruments such as the ADOS-G [56] and the Autism Diagnostic Interview (ADI-R [81], placing motor stereotypies and stimming (behaviours such as spinning, flicking or twiddling) into the sensorimotor domain. RRBI is the domain where sex differences are most likely to occur in children and young people [2, 5, 15], with boys showing significantly greater symptomatology. A sex difference in self-reported symptoms in adulthood seems to contradict this finding, but the non-equivalence of ‘circumscribed interests’ to the RRBI domain makes interpretation somewhat challenging, as RRBI includes sensory and motor abnormalities which we discuss separately below.

In the language domain, an interaction of sex and diagnosis revealed that where normative sex differences appeared between typically developing men and women (perhaps reflecting the commonly held belief of female superiority in communication [82]); there were no statistical differences in the language symptomatology reported by autistic adults. This finding corroborates previous reports of attenuated sex differences in autistic individuals [53–55]. In self-report form, our autistic participants did not corroborate previous suggestions that language skills may be superior in autistic females [5, 21, 24]. We note, however, the rather narrow coverage of the language domain: of these seven items, four relate to literal interpretation of language (e.g. ‘The phrase “I’ve got you under my skin” makes me very uncomfortable’), and only one relates to the ability to engage in reciprocal conversation, which is the area where the camouflage of autistic women serves them well. As such, it is possible that the language measure of the RAADS-R lacks the refinement to pick up a genuine sex difference.

Indeed, at this point, let us further discuss and attempt to interpret the lack of statistical difference between autistic men and women in the social relatedness, circumscribed interests and language domains. The data informs us that autistic women do not rate themselves as significantly more or less symptomatic than men in any one of these domains, but whether these findings reflect a genuine equalisation of childhood differences is equivocal. We have noted, above, the differences that may emerge between studies using self-report data vs. objective observations. A further interpretation of the lack of differentiation seen here and in some other studies of male and female autistics at different ages [11–13] is that it reflects unsuccessful attempts to quantify these differences at domain level [5]. Particularly rich, clinically useful data has come from studies conducting detailed analysis of diagnostic criteria within domains (see Hiller et al. [20] for example). Unfortunately, such scrutiny of individual items was impossible in this meta-analysis where we received only domain scores.

Another interpretation for the lack of differentiation in these domains concerns the diagnostic bottleneck or ‘ascertainment bias’ [5, 15, 22, 35, 57]. This and previous studies of the female autistic phenotype are limited by an inherent selection bias in participants. The current conceptualisation of autism, and the diagnostic tools and screening instruments used to detect it, are undeniably androcentric, being developed and standardised according to male cases. The same can be said of the original and revised Ritvo scale, given the heavy male bias in the original and the standardisation sample. As such, when studies examine diagnosed women who could conceivably more closely match the androcentric symptom presentation defined by the tests, similarities to autistic men may be artificially inflated. Studies have attempted to mitigate this problem in several ways: some have included women who do not reach cut-offs in gold-standard tests but whose diagnoses have been confirmed by experience clinicians [53], whereas others have recruited late-diagnosed women whose growing up undiagnosed suggests they did not fit the archetypal presentation of autism [58]. Nevertheless, the potential exclusion of swathes of less stereotypical autistic women casts a modicum of uncertainty on many findings. Future targets for research may be precisely those women referred for diagnosis whom fail to reach cut off on the androcentric instruments of diagnosis but who fulfil criteria for a developmental social and communication disorder on more dimensional scales, such as the Diagnostic Interview for Social and Communication Disorders (DISCO [83]). Here, differences in cognition, emotion and behaviour not only between autistic men and women but between classically diagnosed and sub-threshold women might be highly illuminative and reveal a broader female spectrum.

An interesting finding of potential import for diagnosis and conceptualization of autism was the divergence between autistic men and women in the sensorimotor domain. This documents hypersensitivity and extraordinarily negative reactions to the textures of foods and clothes, sounds, noises, lights and being touched by others; hyposensitivity to pain and sensation-seeking behaviours like hand-fiddling, rocking or spinning; experiencing the same sensations as variably too intense or not registering them; and movement coordination problems.^1^ Here, a main effect of sex revealed that females generally reported more sensorimotor differences than males, and a main effect of diagnosis corroborated the established sensorimotor abnormalities associated with autism [84, 85]. An interaction of sex and diagnosis revealed, however, that autistic women reported disproportionately more sensorimotor symptoms than their male counterparts. There are extensive reports of sensorimotor abnormalities in autism (see [86, 87] for review), but as usual these are strongly androcentric with few or no female cases. Comparisons with neurotypical peers suggest that motor symptoms are certainly present in autistic girls [88], as are sensory abnormalities [33]; comparisons between males and females on the spectrum, however, are much more scarce. Whilst some imply that sensorimotor abnormalities are equivalent [27, 48], one small study using the Japanese version of the CARS [89] found autistic girls between 5 and 9 years of age to show significantly greater abnormality than autistic boys in their responses to taste, smell and touch, and lesser abnormality in their activity level and bodily movements [49]. Interestingly, women generally obtain higher scores than men on this RAADS-R domain, with autistic women reporting the greatest number of symptoms [70]; the same pattern is seen in the 14-item version of the test [71]. Lai et al. [53] created a composite sensory abnormality score from three items of the ADI-R [81] tapping unusual sensory interests, noise hypersensitivity and extraordinarily negative responses to sensory stimuli. According to caregivers who completed the interview, these items were significantly more prevalent in autistic women.

If sensorimotor abnormalities are indeed a more prevalent feature of female autism than, say, the stereotypical manifestation of repetitive and restricted interests [15, 17, 24, 42–45], this finding would have important diagnostic implications. Sensorimotor abnormalities are downplayed in gold-standard diagnostic tests such as the ADOS-G and the ADI-R, which could, in this context, bias the tests away from detecting females. The suggestion must, however, be treated with caution, based as it is on one study with a small sample [49] and one with a measure lacking sensitivity to sensorimotor abnormalities [53]. It has been proposed that autistic women may have greater capacity for self-reflective awareness in symptom reporting [1, 53], although in our study they did not rate themselves more symptomatic than men in other domains. In line with the general sex difference in the RAADS-R sensorimotor domain [70], some studies suggest that women are generally more likely to report symptoms they perceive as abnormal and indeed to utilise medical services [90, 91], and this may be a normative sex difference that exists in both autistic and non-autistic people. As such, the particular focus that autistic women place on sensorimotor symptoms should be validated by independent, objective measures to investigate whether it has a basis in fact. ‘Sensory subtypes’ have recently been proposed in childhood autism, although gender did not appear to modulate a child’s sensory profile [92]. With a sample of 203 boys and 25 girls in this study, however, this might be worth investigating in a more balanced child and adult sample.

Alongside the avenues for future research suggested by our findings, the nature of the present study leaves several limitations on which further work could build. Primarily, although we were able to obtain a large dataset from other researchers to compliment the data we obtained from local clinics, we received only scores for each domain (social relatedness, circumscribed interests, language and sensorimotor) as a whole. This lack of scores for individual items *within* domains precluded other types of analysis, such as those exploring the factor structure of the RAADS-R and potential differences in the same between males and females. The original authors did not focus on sex differences and so reported a factor structure from a heavily male-dominated sample. Notably, however, they did report the emergence of a different factor than the sensorimotor one that remains in popular usage of the test: a factor identified as social anxiety. Lacking access to the scores to individual items, we were unable to calculate scores in this alternative domain for our male and female participants—however, it seems highly possible that social anxiety is an area where autistic males and females might diverge, given the suggested greater social motivation of autistic females [29, 30].

It is furthermore important to consider the potential influence of several variables which we were unable to control for in the present analysis. Firstly, we unfortunately lacked information regarding psychiatric comorbidities and even additional neurodevelopmental conditions (such as ADHD) in our participants. Whilst neurological conditions were controlled to an extent in some of the data we obtained, we were not privy to information regarding psychiatric comorbidities in any of the participants, thus precluding a more refined analysis. We thus cannot speculate on the effects of psychiatric comorbidities on responses to the RAADS-R (furthermore, we note with interest that the original authors did not appear to screen out additional psychiatric comorbidities in their standardisation sample). This may be highly important, given the greatly elevated prevalence of mental illness in ASC [93], and indeed the high likelihood of autistic females to be misdiagnosed with psychiatric conditions or to come to the attention of clinicians due to other conditions [33].

Race and ethnicity, socioeconomic status and education are other important variables which we were unable to control for in our multi-dataset analysis and which may affect responses to the RAADS-R. The RAADS-R was developed and standardised in Western populations. Although we cannot ascertain the precise ethnicity of each of our participants, it can be surmised with high probability that they were predominantly Caucasian, based on the ethnic diversity of the areas where they were recruited. Alongside sex, these are variables which can notably affect symptom presentation and the likelihood of obtaining an ASC diagnosis. In the UK, age of diagnosis is on average earlier in children with highly educated parents from higher socioeconomic backgrounds [94]; in America, autism diagnoses are substantially higher in the higher socioeconomic groups [95–97]. These statistics are explained largely (although not entirely) by another kind of bottleneck or bias in the diagnostic services: that highly educated parents with greater incomes are more likely and more *able* to approach clinicians with concerns, since many low-income families will lack access to these specialised services. There is, of course, a strong relationship between ethnicity and socioeconomic status. Autistic people from ethnic minority groups are also later to be diagnosed [97–99] and less likely to be diagnosed [97, 100], despite one report of more severe language symptoms in autistic toddlers from minority groups. Culture influences both the manifestation of autistic symptoms [101, 102] and their interpretation by parents and other observers [103]. As is typical of autism research in general, the majority of work in this area focuses on children: much less is known about how these variables affect symptom presentation and the likelihood of obtaining a diagnosis in adulthood, and whether they interact with sex, reflecting a clear need for future study.

As concerns sex, genetic evidence suggests a very real possibility that autism may not be *equally* prevalent in males and females [2–6, 104, 105]. Nevertheless, given their indubitable existence, it is imperative that investigation of the female autistic phenotype remains a high priority, given the suffering reported by late-diagnosed individuals [58, 63]. Maintaining the visibility of this topic is necessary to disseminate this kind of research to professionals within and outside the healthcare fields. A recent, startling finding from Hiller et al. [24] was that the majority of school-age autistic boys had been flagged up by their teachers in the pre-school years, whereas children who had never been a cause for concern were 13 times more likely to be female. The current study furthers investigation of how sex differences present in adulthood, through one screening instrument, the Ritvo Autism Asperger Diagnostic Scale [65]). Our inclusion of a large sample is a strength of the study, but it leaves many openings for future research which should control for psychiatric comorbidities and intellectual function. We may assume from the recruitment techniques and the samples collected (see Additional file 1) that our participants were of average to above-average intelligence. However, the findings cannot speak to the more nuanced issue of how intellectual disability might affect sex differences in autistic symptomatology in adults, and our discussion speaks only to symptom presentation in high-functioning individuals who had completed a self-report measure.

## Conclusions

In these high-functioning individuals, the data from our meta-analysis reveals that autistic women did not statistically differ from autistic men in self-reported symptomatology in domains related to social-relatedness, language and circumscribed interest, but should be ratified by objective measures. It also highlights again the need for research to take into account the so-called ascertainment bias in studying those women who have reached diagnostic cut-offs on androcentric measures, and so whom may plausibly display a more male-like profile. Given the frequent use of child, adolescent and adult screening instruments by diagnostic services, whether these tools can adequately detect more unusual female presentations and subtle camouflaging, as implied in the qualitative literature [34, 35, 58], is of serious concern. An emergent emphasis by autistic women on their sensorimotor symptoms, however, is of potential clinical relevance, given the traditional downplaying of these items in diagnostic instruments and criteria, and requires further investigation.

## Additional file

Additional file 1: Details of participant cohorts, meta-analysis procedure. (DOCX 56 kb)

## Abbreviations

ADI-R: Autism Diagnostic Interview Revised
ADOS-G: Autism Diagnostic Observation Schedule
ASC: Autism spectrum conditions
RAADS-R: Ritvo Autism Asperger Diagnostic Scale Revised
RRBI: Repetitive and restricted behaviours and interest
TD: Typically developing

## Footnotes

1 Unusually, four of the twenty items within this domain describe difficulties modulating vocal tone, pitch and volume which in other diagnostic instruments are related rather to differences in language and social communication [56, 81]. The apparent incongruence of these items within this domain is corroborated by the fact that the factor analysis conducted by the original authors [65] found these items loaded instead on factors related to social relatedness and social blindness. The sensorimotor domain, inadequately specific for autism, was subsequently removed from the RAADS-14 [71]
