# Supplementary material for: Self-reported sex differences in high-functioning adults with autism: a meta-analysis
Source: Mol Autism. 2018 May 18;9:33. doi: 10.1186/s13229-018-0216-6 (PMC5960195; doi:10.1186/s13229-018-0216-6)
Supplement: Supplementary file 1 — Details of participant cohorts, meta-analysis procedure. (DOCX 56 kb) [file 13229_2018_216_MOESM1_ESM.docx]

**Additional file**

**Supplementary Materials 1: Details of participant cohorts**

We obtained datasets from several (but not all) of the sources we contacted. Due to an abundance of typically-developed females and some queried datasets, not all participants were included in the analysis reported in the main text. Here we provide details of the cohorts we collected.

- *Bournemouth University*

The ASC sample obtained by the authors at Bournemouth University consisted of 42 autistic women (mean age = 34.4 [SD: 10.85]) and 42 age-matched autistic men (mean age = 34.25 [SD: 10.76]). These participants were individuals who had presented for and received a diagnosis of Asperger Syndrome at the Dorset NHS Community Adult Asperger Service (CAAS) between 2012-2015. Although IQ testing was not conducted, participants were individuals who had gone undiagnosed until adulthood, so should be assumed to be of average or above-average IQ (> 70). Comorbid conditions, such as anxiety or depression, were not recorded.

Prior to the study being conducted, the CAAS clinic had obtained ethical approval from the NHS in order to conduct the RAADS-R on individuals who presented for diagnosis, who had been referred to the clinic from their general practitioner. Participants volunteered to complete this additional assessment. Collaboration between the clinic and the authors at Bournemouth University allowed us to access the mean scores of each participant in the four domains of the RAADS-R, although we did not have access to scores on individual items. Nor did we have access to any personal or identifying information. A confidentiality agreement was signed between CAAS and the authors, which specified that data would not be shared.

We also obtained a sample of typically-developing, non-autistic participants who were recruited on a voluntary basis from the local Bournemouth area. We excluded those who had reported that they had a current or historic psychiatric illness. The respondents were mainly students, and we noticed the presence of several individuals with scores considerably higher than the suggested RAADS-R cut-off point for ASC (65). We had screened participants for diagnosed autism spectrum conditions, but to avoid the possibility of undiagnosed individuals with autism in the control group, we implemented an exclusion criteria for participants who scored 10% over the RAADS-R cut off point (i.e. participants who scored over 71.5 in the RAADS-R total). We therefore excluded 28 of 58 control males and 23 of 131 control females. This is 56% and 17.6% of the overall collected numbers for these groups respectively. It was surprising to see such a high distribution of males scoring above 71.5 in the RAADS-R total, a percentage that does not appear to align with the suggested prevalence of ASC in the population [1]. It may be worth mentioning that the sample were predominantly Psychology students, which is why females outnumbered males so strongly. We query whether males are perhaps less interested in this topic unless they have specific reason to be. This is however conjecture and we would not claim that a higher than average proportion of autistic individuals enter Psychology, although they are indeed known to be drawn to the STEM topics [2] and STEM careers are generally associated with a higher than average number of autistic traits [3].

- *The Libero/Kana group, MIND Institute of Sacramento, California / University of Alabama at Birmingham*

We received a sample of 19 individuals with ASC from this group: five women (mean age: 34 [SD: 7]) and 14 men (mean age: 30.6 [SD: 8]). This group provided IQ scores for their sample: the ASC group had a mean full-scale IQ of 115.5 (SD: 12.6) as measured by the Wechsler Abbreviated Scale of Intelligence [4], and no individuals fell below a cut-off of 80. All participants had been formally diagnosed through use of the Autism Diagnostic Interview-Revised (ADI-R [5]) and the Autism Diagnostic Observation Schedule (ADOS [6]): the authors did not perform the diagnostic assessment but verified the diagnosis in the participants’ clinical records. ASC participants were recruited from clinics and service providers in Alabama, USA.

We also received 17 control participants, four women (mean age: 33.4 [SD: 10]) and 13 men (mean age: 38.8 [SD: 12.8]), who together had an average full-scale IQ of 118 (SD: 10.4). These participants were screened through a self-report history questionnaire to exclude individuals with neuropsychiatric disorders (ASC, ADHD, Tourettes) and were medication-free. They were volunteers recruited from the Birmingham, Alabama area.

Data from both participant groups was reported in:

Libero, L. E., Maximo, J. O., Deshpande, H. D., Klinger, L. G., Klinger, M. R., & Kana, R. K. (2014). The role of mirroring and mentalizing networks in mediating action intentions in autism. *Molecular Autism, 5*(1), 50.

Libero, L. E., DeRamus, T. P., Lahti, A. C., Deshpande, G., & Kana, R. K. (2015). Multimodal neuroimaging based classification of autism spectrum disorder using anatomical, neurochemical, and white matter correlates. *Cortex, 66*, 46-59.

Libero, L. E., Reid, M. A., White, D. M., Salibi, N., Lahti, A. C., & Kana, R. K. (2016). Biochemistry of the cingulate cortex in autism: An MR spectroscopy study. *Autism Research, 9*(6), 643-657.

Libero, L. E., Burge, W. K., Deshpande, H. D., Pestilli, F., & Kana, R. K. (2016). White Matter Diffusion of Major Fiber Tracts Implicated in Autism Spectrum Disorder. *Brain Connectivity, 6*(9), 691-699.

- *The Kirkovski/Fitzgerald group, Monash University, Australia / Deakin University, Australia*

From this group we received a sample of 13 autistic women (mean age: 26.7 [SD: 7.6]) and 12 autistic men (mean age: 33.9 [SD: 9.2]). These individuals had been formally diagnosed by an experience clinician (psychiatric, psychologist or paediatrician) prior to the study they took part in, with the authors verifying the diagnosis through access to each participant’s diagnostic report. All but four of the participants had a diagnosis of Asperger Syndrome (three women and a man were diagnosed with high-functioning autism). We were able to access intelligence scores as measured by the Kaufman Brief Intelligence Test second edition (KBIT-2 [7]): autistic participants as a group showed an average composite (verbal + non-verbal) IQ score of 109.8 (SD: 14.9).

We also received 12 female controls (mean age: 27.4 [SD: 10.3]) and 12 male controls (mean age: 33.1 [SD: 9.4]), who showed an average IQ composite score of 113.3 (SD: 13.6). Both autistic and non-autistic participants were screened for a history of intellectual disability, psychiatric or neurological disorder, but autistic participants with anxiety and mood disorders were not excluded due to the extremely high incidence of these conditions in this population.

Participants from each group were recruited from via flyers placed around university campus, social media (facebook/gumtree), presentations at support groups for ASD, and the Monash Alfred Psychiatry Research Centre (MAPrc) participant database.

Data from these participants was reported in:

Kirkovski, M., Enticott, P. G., Maller, J. J., Rossell, S. L., & Fitzgerald, P. B. (2015). Diffusion tensor imaging reveals no white matter impairments among adults with autism spectrum disorder. *Psychiatry Research: Neuroimaging, 233*(1), 64-72.

Kirkovski, M., Enticott, P. G., Hughes, M. E., Rossell, S. L., & Fitzgerald, P. B. (2016). Atypical neural activity in males but not females with autism spectrum disorder. *Journal of Autism and Developmental Disorders, 46*(3), 954-963.

Kirkovski, M., Rogasch, N. C., Saeki, T., Fitzgibbon, B. M., Enticott, P. G., & Fitzgerald, P. B. (2016). Single pulse transcranial magnetic stimulation-electroencephalogram reveals no electrophysiological abnormality in adults with high-functioning autism spectrum disorder. *Journal of Child and Adolescent Psychopharmacology, 26*(7), 606-616.

- *Schwartzman / Kapp group, University of California, Los Angeles, USA*

We received a sample of 152 individuals with a formally diagnosed ASC from this group. 11 individuals whose sex was reported as ‘other’ were discarded, such that 141 participants with ASC were suitable for use in the meta-analysis: 84 women (mean age: 36.8 [SD: 13.5]), and 57 men (mean age: 36.3 [13.6]). This was a volunteer sample who responded to advertisements in the local area (Los Angeles), online (autism-related websites, forums and blogs) and on social media sites, and to emails sent to autism support groups and centres world-wide (this was an online study). These authors also included participants with self-diagnosed ASC and with elevated autistic traits in their study, but we include only those who reported having a formal diagnosis, though no more details are had regarding this. All but 11 (7%) scored above the RAADS-R suggested cut-off of 65, but Ritvo and colleagues themselves recognised the possibility of autistic individuals falling below cut-off.

This group also supplied us with data from 479 control participants without a formal diagnosis of ASC, who did not consider themselves to be on the spectrum. As there were two participants here who marked their sex as ‘other’, this left 477 participants: 381 female controls (mean age: 36.2 [SD: 13.2]) and 96 male controls (mean age: 35.8 [SD: 14]).

No details are had regarding IQ of either group, but in the original Schwartzman study, these autistic and non-autistic individuals were part of a larger group (n = 828, which also included, for example, self-diagnosed individuals with ASC and those uncertain if they felt they were on the autism spectrum) of whom 92% (761) had been to college, so we may assume that participants were of average to above-average IQ and did not have an intellectual disability. Schwartzman et al report that of these 828 participants, 80% were Caucasian, 5% Asian, 4.5% Hispanic, 2% of African descent, 2% Middle Eastern, 5% multi-ethnic, and 1.5% declining to answer. Respondents came from 31 different countries but 84% of participants were from the United States.

Unlike the Libero/Kana and the Kirkovski/Fitzgerald group, and like our own data, Schwartzman et al. did not screen their control participants for the presence of an undiagnosed ASC. As such, as with our own dataset, we removed control participants who scored over 10% higher than the 65 RAADS-R suggested cut-off for autism (i.e. participants who scored over 71.5 [72 and above] in the RAADS-R total). Removing 41 control women and 14 control men left us with 340 control women (mean age: 36.3 [SD: 13.2]) and 82 control men (mean age: 35.9 [SD: 14]). This is 10.8% and 14.6% removed of the total number of control men and women originally.

Data from these participants was originally reported in:

Schwartzman, B. C., Wood, J. J., & Kapp, S. K. (2016). Can the Five Factor Model of Personality Account for the Variability of Autism Symptom Expression? Multivariate Approaches to Behavioral Phenotyping in Adult Autism Spectrum Disorder. *Journal of Autism and Developmental Disorders, 46*(1), 253-272.

**Supplementary Material 2: Database search (Step 1 of Meta-Analysis)**

Relevant studies are displayed in italics.

*Web of Science* search results (6):

1. Vagni, D., Moscone, D., Travaglione, S., & Cotugno, A. (2016). Using the Ritvo Autism Asperger Diagnostic Scale-Revised (RAADS-R) disentangle the heterogeneity of autistic traits in an Italian eating disorder population. Research in Autism Spectrum Disorders, 32, 143-155.
2. *Sizoo, B. B., Horwitz, E. H., Teunisse, J. P., Kan, C. C., Vissers, C. T. W. M., Forceville, E. J. M., ... & Geurts, H. M. (2015). Predictive validity of self-report questionnaires in the assessment of autism spectrum disorders in adults. Autism, 19(7), 842-849.*
3. Eriksson, J. M., Andersen, L. M., & Bejerot, S. (2013). RAADS-14 Screen: validity of a screening tool for autism spectrum disorder in an adult psychiatric population. Molecular Autism, 4(1), 49.
4. *Andersen, L. M., Näswall, K., Manouilenko, I., Nylander, L., Edgar, J., Ritvo, R. A., ... & Bejerot, S. (2011). The Swedish version of the Ritvo autism and Asperger diagnostic scale: Revised (RAADS-R). A validation study of a rating scale for adults. Journal of autism and developmental disorders, 41(12), 1635-1645.*
5. *Ritvo, R. A., Ritvo, E. R., Guthrie, D., Ritvo, M. J., Hufnagel, D. H., McMahon, W., ... & Eloff, J. (2011). The Ritvo Autism Asperger Diagnostic Scale-Revised (RAADS-R): a scale to assist the diagnosis of Autism Spectrum Disorder in adults: an international validation study. Journal of autism and developmental disorders, 41(8), 1076-1089.*
6. Stoesz, B. M., Montgomery, J. M., Smart, S. L., & Hellsten, L. A. M. (2011). Review of five instruments for the assessment of Asperger's disorder in adults. The Clinical Neuropsychologist, 25(3), 376-401.

*PubMed* search results (4):

1. *Sizoo, B. B., Horwitz, E. H., Teunisse, J. P., Kan, C. C., Vissers, C. T. W. M., Forceville, E. J. M., ... & Geurts, H. M. (2015). Predictive validity of self-report questionnaires in the assessment of autism spectrum disorders in adults. Autism, 19(7), 842-849.*
2. Eriksson, J. M., Andersen, L. M., & Bejerot, S. (2013). RAADS-14 Screen: validity of a screening tool for autism spectrum disorder in an adult psychiatric population. Molecular Autism, 4(1), 49.
3. *Andersen, L. M., Näswall, K., Manouilenko, I., Nylander, L., Edgar, J., Ritvo, R. A., ... & Bejerot, S. (2011). The Swedish version of the Ritvo autism and Asperger diagnostic scale: Revised (RAADS-R). A validation study of a rating scale for adults. Journal of autism and developmental disorders, 41(12), 1635-1645.*
4. *Ritvo, R. A., Ritvo, E. R., Guthrie, D., Ritvo, M. J., Hufnagel, D. H., McMahon, W., ... & Eloff, J. (2011). The Ritvo Autism Asperger Diagnostic Scale-Revised (RAADS-R): a scale to assist the diagnosis of Autism Spectrum Disorder in adults: an international validation study. Journal of autism and developmental disorders, 41(8), 1076-1089.*

*Google Scholar* search results (85):

** indicates that study did not use our specific version of the RAADS-R but rather the revised 14-item version or the original scale.

1. Lord, C., Corsello, C., & Grzadzinski, R. (2014). Diagnostic instruments in autistic spectrum disorders. Handbook of Autism and Pervasive Developmental Disorders, Fourth Edition.
2. Falkmer, T., Anderson, K., Falkmer, M., & Horlin, C. (2013). Diagnostic procedures in autism spectrum disorders: a systematic literature review. European child & adolescent psychiatry, 22(6), 329-340.
3. **Mathersul, D., McDonald, S., & Rushby, J. A. (2013). Understanding advanced theory of mind and empathy in high-functioning adults with autism spectrum disorder. Journal of Clinical and Experimental Neuropsychology, 35(6), 655-668.
4. *Schneider, D., Slaughter, V. P., Bayliss, A. P., & Dux, P. E. (2013). A temporally sustained implicit theory of mind deficit in autism spectrum disorders. Cognition, 129(2), 410-417.*
5. *Andersen, L. M., Näswall, K., Manouilenko, I., Nylander, L., Edgar, J., Ritvo, R. A., ... & Bejerot, S. (2011). The Swedish version of the Ritvo autism and Asperger diagnostic scale: Revised (RAADS-R). A validation study of a rating scale for adults. Journal of autism and developmental disorders, 41(12), 1635-1645.*
6. **Mathersul, D., McDonald, S., & Rushby, J. A. (2013). Psychophysiological correlates of social judgement in high-functioning adults with autism spectrum disorder. International Journal of Psychophysiology, 87(1), 88-94.
7. Eriksson, J. M., Andersen, L. M., & Bejerot, S. (2013). RAADS-14 Screen: validity of a screening tool for autism spectrum disorder in an adult psychiatric population. Molecular Autism, 4(1), 49.
8. **Mathersul, D., McDonald, S., & Rushby, J. A. (2013). Automatic facial responses to affective stimuli in high-functioning adults with autism spectrum disorder. Physiology & behavior, 109, 14-22.
9. Mandell, D. S., Lawer, L. J., Branch, K., Brodkin, E. S., Healey, K., Witalec, R., ... & Gur, R. E. (2012). Prevalence and correlates of autism in a state psychiatric hospital. Autism, 16(6), 557-567.
10. Stoesz, B. M., Montgomery, J. M., Smart, S. L., & Hellsten, L. A. M. (2011). Review of five instruments for the assessment of Asperger's disorder in adults. The Clinical Neuropsychologist, 25(3), 376-401.
11. Lai, M. C., & Baron-Cohen, S. (2015). Identifying the lost generation of adults with autism spectrum conditions. The Lancet Psychiatry, 2(11), 1013-1027.
12. Lau, W. Y. P., Kelly, A. B., & Peterson, C. C. (2013). Further evidence on the factorial structure of the autism spectrum quotient (AQ) for adults with and without a clinical diagnosis of autism. Journal of autism and developmental disorders, 43(12), 2807-2815.
13. **Mathersul, D., McDonald, S., & Rushby, J. A. (2013). Automatic facial responses to briefly presented emotional stimuli in autism spectrum disorder. Biological psychology, 94(2), 397-407.
14. Brugha, T. S., Doos, L., Tempier, A., Einfeld, S., & Howlin, P. (2015). Outcome measures in intervention trials for adults with autism spectrum disorders; a systematic review of assessments of core autism features and associated emotional and behavioural problems. International journal of methods in psychiatric research, 24(2), 99-115.
15. *Libero, L. E., Maximo, J. O., Deshpande, H. D., Klinger, L. G., Klinger, M. R., & Kana, R. K. (2014). The role of mirroring and mentalizing networks in mediating action intentions in autism. Molecular autism, 5(1), 50.*
16. *Kirkovski, M., Enticott, P. G., Maller, J. J., Rossell, S. L., & Fitzgerald, P. B. (2015). Diffusion tensor imaging reveals no white matter impairments among adults with autism spectrum disorder. Psychiatry Research: Neuroimaging, 233(1), 64-72.*
17. *Libero, L. E., DeRamus, T. P., Lahti, A. C., Deshpande, G., & Kana, R. K. (2015). Multimodal neuroimaging based classification of autism spectrum disorder using anatomical, neurochemical, and white matter correlates. Cortex, 66, 46-59.*
18. Sappok, T., Heinrich, M., & Underwood, L. (2015). Screening tools for autism spectrum disorders. Advances in Autism, 1(1), 12-29.
19. Takei, R., Matsuo, J., Takahashi, H., Uchiyama, T., Kunugi, H., & Kamio, Y. (2014). Verification of the utility of the social responsiveness scale for adults in non-clinical and clinical adult populations in Japan. BMC psychiatry, 14(1), 302.
20. Lerman, D. C., Hawkins, L., Hoffman, R., & Caccavale, M. (2013). TRAINING ADULTS WITH AN AUTISM SPECTRUM DISORDER TO CONDUCT DISCRETE‐TRIAL TRAINING FOR YOUNG CHILDREN WITH AUTISM: A PILOT STUDY. Journal of applied behavior analysis, 46(2), 465-478.
21. Brosnan, M., Lewton, M., & Ashwin, C. (2016). Reasoning on the autism spectrum: a dual process theory account. Journal of autism and developmental disorders, 46(6), 2115-2125.
22. *Manouilenko, I., Pagani, M., Stone-Elander, S., Odh, R., Brolin, F., Hatherly, R., ... & Bejerot, S. (2013). Autistic traits, ADHD symptoms, neurological soft signs and regional cerebral blood flow in adults with autism spectrum disorders. Research in Autism Spectrum Disorders, 7(5), 566-578.*
23. *Kirkovski, M., Enticott, P. G., Hughes, M. E., Rossell, S. L., & Fitzgerald, P. B. (2016). Atypical neural activity in males but not females with autism spectrum disorder. Journal of autism and developmental disorders, 46(3), 954-963.*
24. Ratner, K., & Berman, S. L. (2015). The influence of autistic features on identity development in emerging adults. Emerging Adulthood, 3(2), 136-139.
25. Grodberg, D., Weinger, P. M., Halpern, D., Parides, M., Kolevzon, A., & Buxbaum, J. D. (2014). The autism mental status exam: sensitivity and specificity using DSM-5 criteria for autism spectrum disorder in verbally fluent adults. Journal of autism and developmental disorders, 44(3), 609-614.
26. Simoncini, M., Miniati, M., Vanelli, F., Callari, A., Vannucchi, G., Mauri, M., & Dell’Osso, L. (2014). Lifetime autism spectrum features in a patient with a psychotic mixed episode who attempted suicide. Case reports in psychiatry, 2014.
27. Carpenter, P. (2012). Diagnosis and assessment in autism spectrum disorders. Advances in Mental Health and Intellectual Disabilities, 6(3), 121-129.
28. Pantelis, P. C., & Kennedy, D. P. (2016). Estimation of the prevalence of autism spectrum disorder in South Korea, revisited. Autism, 20(5), 517-527.
29. Westwood, H., Mandy, W., & Tchanturia, K. (2017). Clinical evaluation of autistic symptoms in women with anorexia nervosa. Molecular Autism, 8(1), 12.
30. Eriksson, J. M., Andersen, L. M., & Bejerot, S. (2014). Assessing adults with normal intelligence for ASD. In Comprehensive guide to autism (pp. 369-385). Springer New York.
31. Werner, J. M. (2013). Structural and functional neural correlates of developmental dyspraxia in the mirror neuron system. University of Southern California.
32. Soleimani, F., Khakshour, A., Abbasi, Z., Khayat, S., Ghaemi, S. Z., & Hajikhani Golchin, N. A. (2014). Review of autism screening tests. International Journal of Pediatrics, 2(4.1), 319-329.
33. Howlin, P., Arciuli, J., Begeer, S., Brock, J., Clarke, K., Costley, D., ... & Guastella, A. (2015). Research on adults with autism spectrum disorder: Roundtable report. Journal of Intellectual and Developmental Disability, 40(4), 388-393.
34. Brewer, N., & Young, R. L. (2015). Crime and Autism Spectrum Disorder: Myths and Mechanisms. Jessica Kingsley Publishers.
35. *Zimmerman, D. L., Ownsworth, T., O'Donovan, A., Roberts, J., & Gullo, M. J. (2016). Independence of hot and cold executive function deficits in high-functioning adults with autism spectrum disorder. Frontiers in human neuroscience, 10.*
36. Wilson, C. E., Roberts, G., Gillan, N., Ohlsen, C., Robertson, D., & Zinkstok, J. (2013). The NICE guideline on recognition, referral, diagnosis and management of adults on the autism spectrum. Advances in Mental Health and Intellectual Disabilities, 8(1), 3-14.
37. *Sizoo, B. B., Horwitz, E. H., Teunisse, J. P., Kan, C. C., Vissers, C. T. W. M., Forceville, E. J. M., ... & Geurts, H. M. (2015). Predictive validity of self-report questionnaires in the assessment of autism spectrum disorders in adults. Autism, 19(7), 842-849.*
38. BEJEROT, S., & NORDIN, V. (2014). Autismspektrumsyndrom ersätter Aspergers syndrom och autism. Lakartidningen, 111.
39. Satterfield, D., Lepage, C., & Ladjahasan, N. (2015). Preferences for online course delivery methods in higher education for students with autism spectrum disorders. Procedia Manufacturing, 3, 3651-3656.
40. Westwood, H., Mandy, W., Simic, M., & Tchanturia, K. (2017). Assessing ASD in adolescent females with anorexia nervosa using clinical and developmental measures: a preliminary investigation. Journal of Abnormal Child Psychology, 1-10.
41. Rutherford, M., McKenzie, K., McClure, I., Forsyth, K., O’Hare, A., McCartney, D., & Finlayson, I. (2016). A national study to investigate the clinical use of standardised instruments in autism spectrum disorder assessment of children and adults in Scotland. Research in Autism Spectrum Disorders, 29, 93-100.
42. Rench, C. (2014). When Eros meets autos: Marriage to someone with autism spectrum disorder (Doctoral dissertation, Capella University).
43. Soendergaard, H. M., Thomsen, P. H., Pedersen, P., Pedersen, E., Poulsen, A. E., Nielsen, J. M., ... & Soegaard, H. J. (2016). Treatment dropout and missed appointments among adults with attention-deficit/hyperactivity disorder: associations with patient-and disorder-related factors. The Journal of clinical psychiatry, 77(2), 232-239.
44. Ritvo, A. R. (2013). RAADS-R. In Encyclopedia of Autism Spectrum Disorders (pp. 2489-2490). Springer New York.
45. Horwitz, E. H., Schoevers, R. A., Ketelaars, C. E. J., Kan, C. C., van Lammeren, A. M. D. N., Meesters, Y., ... & Bartels, A. A. J. (2016). Clinical assessment of ASD in adults using self-and other-report: Psychometric properties and validity of the Adult Social Behavior Questionnaire (ASBQ). Research in Autism Spectrum Disorders, 24, 17-28.
46. *Kirkovski, M., Rogasch, N. C., Saeki, T., Fitzgibbon, B. M., Enticott, P. G., & Fitzgerald, P. B. (2016). Single pulse transcranial magnetic stimulation-electroencephalogram reveals no electrophysiological abnormality in adults with high-functioning autism spectrum disorder. Journal of child and adolescent psychopharmacology, 26(7), 606-616.*
47. Satterfield, D., Lepage, C., & Ladjahasan, N. (2015, August). Presence of Autism Spectrum Disorders in University Students: Implications for Education and HCI. In International Conference on Universal Access in Human-Computer Interaction (pp. 681-688). Springer International Publishing.
48. Carrington, S. J. (2016). Implications of ICD and DSM on Screening, Diagnosis, and Monitoring. In Handbook of Assessment and Diagnosis of Autism Spectrum Disorder (pp. 117-136). Chicago: Springer International Publishing.
49. Eriksson, J. (2016). Autism spectrum disorder beyond the extreme male brain.
50. Zimmerman, D., Ownsworth, T., O’Donovan, A., Roberts, J., & Gullo, M. J. PhD Spotlight. AUSTRALIAN, 30.
51. *Libero, L. E., Reid, M. A., White, D. M., Salibi, N., Lahti, A. C., & Kana, R. K. (2015). Biochemistry of the cingulate cortex in autism: An MR spectroscopy study. Autism Research.*
52. Brock, J. Research on Adults with Autism–Roundtable Report.
53. Statham, D. (2015). Autism Assessment Measures. Australian Clinical Psychologist, 1(2), 11-13.
54. *Sizoo, B., & Horwitz, E. (2014). EPA-0548-Assessment of the psychometric properties of the raads-r and two abridged versions of the autism spectrum quotient (AQ-10 and AQ-28) in a dutch adult outpatient population. European Psychiatry, 29, 1.*
55. van Wijngaarden-Cremers, P. J., & van der Gaag, R. J. (2015). Addiction and Autism Spectrum Disorder. In Co-occurring Addictive and Psychiatric Disorders (pp. 193-204). Springer Berlin Heidelberg.
56. Magiati, I. (2016). Assessment in Adulthood. In Handbook of Assessment and Diagnosis of Autism Spectrum Disorder (pp. 191-207). Springer International Publishing.
57. *Schwartzman, B. C., Wood, J. J., & Kapp, S. K. (2016). Can the Five Factor Model of Personality Account for the Variability of Autism Symptom Expression? Multivariate Approaches to Behavioral Phenotyping in Adult Autism Spectrum Disorder. Journal of autism and developmental disorders, 46(1), 253-272.*
58. Caruana, N., Stieglitz Ham, H., Brock, J., Woolgar, A., Kloth, N., Palermo, R., & McArthur, G. (2017). Joint attention difficulties in autistic adults: An interactive eye-tracking study. Autism, 1362361316676204.
59. Dell'Osso, L., Gesi, C., Massimetti, E., Cremone, I. M., Barbuti, M., Maccariello, G., ... & Bossini, L. (2017). Adult Autism Subthreshold Spectrum (AdAS Spectrum): Validation of a questionnaire investigating subthreshold autism spectrum. Comprehensive Psychiatry, 73, 61-83.
60. Close, M. PUBLISH OR PUBLISH.
61. Gook, L. (2014). Offenders with autism spectrum disorder: screening, characteristics and staff awareness (No. Doctor of Philosophy (Forensic)). Deakin University.
62. Libero, L. E. (2012). The Role of the Mirror Neuron System in Mental State Attribution in Autism: An FMRI Study (Doctoral dissertation, University of Alabama at Birmingham).
63. Russet, F., Pernon, E., Fiard, D., & Baghdadli, A. (2016). De la nécessité pour les équipes francophones de disposer d’outils d’aide au diagnostic des troubles du spectre autistique chez l’adulte sans déficience intellectuelle. L'Encéphale, 42(6), 598-599.
64. *Libero, L. E., Burge, W. K., Deshpande, H. D., Pestilli, F., & Kana, R. K. (2016). White Matter Diffusion of Major Fiber Tracts Implicated in Autism Spectrum Disorder. Brain Connectivity, 6(9), 691-699.*
65. Mateescu, L., Mihailescu, I., Frunza, A. A., Coman, M., Rad, F., Anghel, C. G., ... & Manea, M. ASSESSMENT TOOLS FOR AUTISM SPECTRUM DISORDERS IN ADULTS PATIENTS.
66. Zimmerman, D., Ownsworth, T., O’Donovan, A., Roberts, J., & Gullo, M. J. (2017). Associations between executive functions and mental health outcomes for adults with autism spectrum disorder. Psychiatry Research, 253, 360-363.
67. *Dunlop, W. A., Enticott, P. G., & Rajan, R. (2016). Speech Discrimination Difficulties in High-Functioning Autism Spectrum Disorder Are Likely Independent of Auditory Hypersensitivity. Frontiers in Human Neuroscience, 10.*
68. Birt, K. (2015). Adult Autism Spectrum Disorder and Intimate Relationships (No. DPsych (Clin)). Deakin Univeristy.
69. Deering, H. J. (2013). Opportunity for success: Website evaluation and scanning by students with Autism Spectrum Disorders.
70. Stoesz, B. M., Montgomery, J. M., & Hellsten, L. the Assessment of Asperger's Disorder in Adults.
71. Keller, R., Piedimonte, A., Bianco, F., Bari, S., & Cauda, F. (2016). Autism Open Access.
72. Baghdadli, A., Russet, F., & Mottron, L. (2017). Measurement properties of screening and diagnostic tools for autism spectrum adults of mean normal intelligence: A systematic review. European Psychiatry.
73. Elwin, M. (2016). Description and measurement of sensory symptoms in autism spectrum (Doctoral dissertation, Örebro university).
74. Satterfield, D., & Kelle, S. (2017). Ethical Issues in Online Education. In Advances in The Human Side of Service Engineering (pp. 257-266). Springer International Publishing.
75. Low, A. Q. Electronic supplementary material. Social Cognition, 62, 12-97.
76. Vagni, D., Moscone, D., Travaglione, S., & Cotugno, A. (2016). Using the Ritvo Autism Asperger Diagnostic Scale-Revised (RAADS-R) disentangle the heterogeneity of autistic traits in an Italian eating disorder population. Research in Autism Spectrum Disorders, 32, 143-155.
77. Stothers, M. E., & Cardy, J. O. (2015). Nonverbal Learning Disabilities and Asperger Syndrome in Young Adults: Vocabulary, Gestalts, and Social Perception. Medical and Educational Perspectives on Nonverbal Learning Disability in Children and Young Adults, 264.
78. Neff, M. R. (2016). Asperger's Syndrome in Adults.
79. Care, P. (2012). Adolescent Health and Development: The Unique Experience of the Adolescent With Asperger’s Syndrome. Nursing of Autism Spectrum Disorder: Evidence-Based Integrated Care Across the Lifespan.
80. Brock, J. Joint attention difficulties in autistic adults: An interactive eye-tracking study.
81. Morgan, P. (2016). Child Protection and Parents with a Learning Disability: Good Practice for Assessing and Working with Adults-including Autism Spectrum Disorders and Borderline Learning Disability. Jessica Kingsley Publishers.
82. Быховский, О. Б. Психологические особенности успешных и неуспешных коммуникативных паттернов.
83. Piana, G., Menighetti, R., De Leonibus, R., Cazzato, S., Apa, M., Cavadi, A., ... & Giostra, N. (2006). sommario.
84. MARTINEZ, G. (2012). DOCTORAT EN MÉDECINE (Doctoral dissertation, UNIVERSITÉ PARIS DESCARTES (PARIS).
85. Bejerot, S. Titel: Replik till Andreas Fries: Ingen tjänar på att det går inflation i autismspektrumbegreppet (jämte) Slutreplik: Såväl över-som underdiagnostik tyder på kunskapsbrist av Andreas Fries 2010 nr 47 sid 2978-9.

*Science Direct* search results (87)

*** indicates that study did not use our specific version of the RAADS-R but rather the revised 14-item version or the original scale.*

1. Vagni, D., Moscone, D., Travaglione, S., & Cotugno, A. (2016). Using the Ritvo Autism Asperger Diagnostic Scale-Revised (RAADS-R) disentangle the heterogeneity of autistic traits in an Italian eating disorder population. Research in Autism Spectrum Disorders, 32, 143-155.
2. Baghdadli, A., Russet, F., & Mottron, L. (2017). Measurement properties of screening and diagnostic tools for autism spectrum adults of mean normal intelligence: A systematic review. European Psychiatry.
3. Satterfield, D., Lepage, C., & Ladjahasan, N. (2015). Preferences for online course delivery methods in higher education for students with autism spectrum disorders. Procedia Manufacturing, 3, 3651-3656.
4. Dell'Osso, L., Gesi, C., Massimetti, E., Cremone, I. M., Barbuti, M., Maccariello, G., ... & Bossini, L. (2017). Adult Autism Subthreshold Spectrum (AdAS Spectrum): Validation of a questionnaire investigating subthreshold autism spectrum. Comprehensive Psychiatry, 73, 61-83.
5. Zimmerman, D., Ownsworth, T., O’Donovan, A., Roberts, J., & Gullo, M. J. (2017). Associations between executive functions and mental health outcomes for adults with autism spectrum disorder. Psychiatry Research, 253, 360-363.
6. Rutherford, M., McKenzie, K., McClure, I., Forsyth, K., O’Hare, A., McCartney, D., & Finlayson, I. (2016). A national study to investigate the clinical use of standardised instruments in autism spectrum disorder assessment of children and adults in Scotland. Research in Autism Spectrum Disorders, 29, 93-100.
7. Lai, M. C., & Baron-Cohen, S. (2015). Identifying the lost generation of adults with autism spectrum conditions. The Lancet Psychiatry, 2(11), 1013-1027.
8. Matson, J. L., & Neal, D. (2009). Diagnosing high incidence autism spectrum disorders in adults. Research in Autism Spectrum Disorders, 3(3), 581-589.
9. Bejerot, S., Eriksson, J. M., & Mörtberg, E. (2014). Social anxiety in adult autism spectrum disorder. Psychiatry research, 220(1), 705-707.
10. Kanai, C., Tani, M., Hashimoto, R., Yamada, T., Ota, H., Watanabe, H., ... & Kato, N. (2012). Cognitive profiles of adults with Asperger's disorder, high-functioning autism, and pervasive developmental disorder not otherwise specified based on the WAIS-III. Research in Autism Spectrum Disorders, 6(1), 58-64.
11. Puig-Alcaraz, C., Fuentes-Albero, M., Calderón, J., Garrote, D., & Cauli, O. (2015). Increased homocysteine levels correlate with the communication deficit in children with autism spectrum disorder. Psychiatry research, 229(3), 1031-1037.
12. Lai, M-C., Lombardo, M. V., & Baron-Cohen, S. (2014). Autism. The Lancet, 383(9920), 896-910.
13. Richdale, A. L., & Schreck, K. A. (2008). Assessment and intervention in autism: An historical perspective. Clinical assessment and intervention for autism spectrum disorders, 3-32.
14. Oberman, L. M., Pascual-Leone, A., & Rotenberg, A. (2016). Chapter 13 – Brain stimulation to understand and modulate the autism spectrum. Pediatric Brain Stimulation: Mapping and Modulating the Developing Brain, 257-279.
15. Muhle, R. A., & Sanders, S. J. (2016). Autism spectrum disorders: genes to pathways to circuits. Genomics, Circuits and Pathways in Clinical Neuropsychology, 443-465.
16. Kuschner, E. S., Eisenberg, I. W., Orionzi, B., Simmons, W. K., Kenworthy, L., Martin, A., & Wallace, G. L. (2015). A preliminary study of self-reported food selectivity in adolescents and young adults with autism spectrum disorder. Research in autism spectrum disorders, 15, 53-59.
17. Tsai, L. Y. (2014). Impact of DSM-5 on epidemiology of autism spectrum disorder. Research in Autism Spectrum Disorders, 8(11), 1454-1470.
18. Alanazi, A. S. (2013). The role of nutraceuticals in the management of autism. Saudi Pharmaceutical Journal, 21(3), 233-243.
19. ** Mathersul, D., McDonald, S., & Rushby, J. A. (2013). Autonomic arousal explains social cognitive abilities in high-functioning adults with autism spectrum disorder. International Journal of Psychophysiology, 89(3), 475-482.
20. **Mathersul, D., McDonald, S., & Rushby, J. A. (2013). Automatic facial responses to affective stimuli in high-functioning adults with autism spectrum disorder. Physiology & behavior, 109, 14-22.
21. Zürcher, N. R., Bhanot, A., McDougle, C. J., & Hooker, J. M. (2015). A systematic review of molecular imaging (PET and SPECT) in autism spectrum disorder: current state and future research opportunities. Neuroscience & Biobehavioral Reviews, 52, 56-73.
22. Kanai, C., Iwanami, A., Hashimoto, R., Ota, H., Tani, M., Yamada, T., & Kato, N. (2011). Clinical characterization of adults with Asperger's syndrome assessed by self-report questionnaires based on depression, anxiety, and personality. Research in Autism Spectrum Disorders, 5(4), 1451-1458.
23. Siegel, M. (2012). Psychopharmacology of autism spectrum disorder: evidence and practice. Child and adolescent psychiatric clinics of North America, 21(4), 957-973.
24. *Sizoo, B., & Horwitz, E. (2014). EPA-0548-Assessment of the psychometric properties of the raads-r and two abridged versions of the autism spectrum quotient (AQ-10 and AQ-28) in a dutch adult outpatient population. European Psychiatry, 29, 1.*
25. *Schneider, D., Slaughter, V. P., Bayliss, A. P., & Dux, P. E. (2013). A temporally sustained implicit theory of mind deficit in autism spectrum disorders. Cognition, 129(2), 410-417.*
26. Rivet, T. T., & Matson, J. L. (2011). Review of gender differences in core symptomatology in autism spectrum disorders. Research in Autism Spectrum Disorders, 5(3), 957-976.
27. Kumazaki, H., Muramatsu, T., Kosaka, H., Fujisawa, T. X., Iwata, K., Tomoda, A., ... & Mimura, M. (2015). Sex differences in cognitive and symptom profiles in children with high functioning autism spectrum disorders. Research in Autism Spectrum Disorders, 13, 1-7.
28. Reilly, C. (2009). Autism spectrum disorders in Down syndrome: A review. Research in Autism Spectrum Disorders, 3(4), 829-839.
29. *Libero, L. E., DeRamus, T. P., Lahti, A. C., Deshpande, G., & Kana, R. K. (2015). Multimodal neuroimaging based classification of autism spectrum disorder using anatomical, neurochemical, and white matter correlates. Cortex, 66, 46-59.*
30. Manouilenko, I., Pagani, M., Stone-Elander, S., Odh, R., Brolin, F., Hatherly, R., ... & Bejerot, S. (2013). Autistic traits, ADHD symptoms, neurological soft signs and regional cerebral blood flow in adults with autism spectrum disorders. Research in Autism Spectrum Disorders, 7(5), 566-578.
31. Canitano, R., & Scandurra, V. (2011). Psychopharmacology in autism: an update. Progress in Neuro-Psychopharmacology and Biological Psychiatry, 35(1), 18-28.
32. Weiner, L., Baratta, A., Henry, J., & Di Santi, C. (2010, December). Apport des approches inspirées de la neuropsychologie au diagnostic d’autisme chez l’adulte: une étude de cas. In Annales Médico-psychologiques, revue psychiatrique (Vol. 168, No. 10, pp. 782-791). Elsevier Masson.
33. Foss-Feig, J. H., Heacock, J. L., & Cascio, C. J. (2012). Tactile responsiveness patterns and their association with core features in autism spectrum disorders. Research in autism spectrum disorders, 6(1), 337-344.
34. Amiet, C., Gourfinkel-An, I., Bouzamondo, A., Tordjman, S., Baulac, M., Lechat, P., ... & Cohen, D. (2008). Epilepsy in autism is associated with intellectual disability and gender: evidence from a meta-analysis. Biological psychiatry, 64(7), 577-582.
35. Chapter 1: History and development of autism spectrum disorders. Assessment and Treatment of Child Psychopathology and Developmental Disabilities, 1, 1-22.
36. Horwitz, E. H., Schoevers, R. A., Ketelaars, C. E. J., Kan, C. C., van Lammeren, A. M. D. N., Meesters, Y., ... & Bartels, A. A. J. (2016). Clinical assessment of ASD in adults using self-and other-report: Psychometric properties and validity of the Adult Social Behavior Questionnaire (ASBQ). Research in Autism Spectrum Disorders, 24, 17-28.
37. Russet, F., Pernon, E., Fiard, D., & Baghdadli, A. (2016). De la nécessité pour les équipes francophones de disposer d’outils d’aide au diagnostic des troubles du spectre autistique chez l’adulte sans déficience intellectuelle. L'Encéphale, 42(6), 598-599.
38. Zwaigenbaum, L., Szatmari, P., Jones, M. B., Bryson, S. E., Maclean, J. E., Mahoney, W. J., ... & Tuff, L. (2002). Pregnancy and birth complications in autism and liability to the broader autism phenotype. Journal of the American Academy of Child & Adolescent Psychiatry, 41(5), 572-579.
39. Girli, A., & Tekin, D. (2010). Investigating false belief levels of typically developed children and children with autism. Procedia-Social and Behavioral Sciences, 2(2), 1944-1950.
40. Soorya, L., Kiarashi, J., & Hollander, E. (2008). Psychopharmacologic interventions for repetitive behaviors in autism spectrum disorders. Child and adolescent psychiatric clinics of North America, 17(4), 753-771.
41. Oristaglio, J., West, S. H., Ghaffari, M., Lech, M. S., Verma, B. R., Harvey, J. A., ... & Malone, R. P. (2013). Children with autism spectrum disorders show abnormal conditioned response timing on delay, but not trace, eyeblink conditioning. Neuroscience, 248, 708-718.
42. Belmonte, M. K., & Carper, R. A. (2006). Monozygotic twins with Asperger syndrome: Differences in behaviour reflect variations in brain structure and function. Brain and Cognition, 61(1), 110-121.
43. Gabriels, R. L., Agnew, J. A., Miller, L. J., Gralla, J., Pan, Z., Goldson, E., ... & Hooks, E. (2008). Is there a relationship between restricted, repetitive, stereotyped behaviors and interests and abnormal sensory response in children with autism spectrum disorders?. Research in Autism Spectrum Disorders, 2(4), 660-670.
44. Gray, K. M., Tonge, B. J., & Brereton, A. V. (2006). Screening for autism in infants, children, and adolescents. International Review of Research in Mental Retardation, 32, 197-227.
45. Stigler, K. A., McDonald, B. C., Anand, A., Saykin, A. J., & McDougle, C. J. (2011). Structural and functional magnetic resonance imaging of autism spectrum disorders. Brain research, 1380, 146-161.
46. Paul, R., Bianchi, N., Augustyn, A., Klin, A., & Volkmar, F. R. (2008). Production of syllable stress in speakers with autism spectrum disorders. Research in autism spectrum disorders, 2(1), 110-124.
47. Posey, D. J., Erickson, C. A., & McDougle, C. J. (2008). Developing drugs for core social and communication impairment in autism. Child and adolescent psychiatric clinics of North America, 17(4), 787-801.
48. Lidstone, J., Uljarević, M., Sullivan, J., Rodgers, J., McConachie, H., Freeston, M., ... & Leekam, S. (2014). Relations among restricted and repetitive behaviors, anxiety and sensory features in children with autism spectrum disorders. Research in Autism Spectrum Disorders, 8(2), 82-92.
49. Tanguay, P. E. (2000). Pervasive developmental disorders: A 10-year review. Journal of the American Academy of Child & Adolescent Psychiatry, 39(9), 1079-1095.
50. Mulder, E. J., Anderson, G. M., Kema, I. P., De Bildt, A., Van Lang, N. D., Den Boer, J. A., & Minderaa, R. B. (2004). Platelet serotonin levels in pervasive developmental disorders and mental retardation: diagnostic group differences, within-group distribution, and behavioral correlates. Journal of the American Academy of Child & Adolescent Psychiatry, 43(4), 491-499.
51. Dominick, K. C., Davis, N. O., Lainhart, J., Tager-Flusberg, H., & Folstein, S. (2007). Atypical behaviors in children with autism and children with a history of language impairment. Research in developmental disabilities, 28(2), 145-162.
52. Levitt, J. G., O'Neill, J., Blanton, R. E., Smalley, S., Fadale, D., McCracken, J. T., ... & Alger, J. R. (2003). Proton magnetic resonance spectroscopic imaging of the brain in childhood autism. Biological psychiatry, 54(12), 1355-1366.
53. Seltzer, M. M., Krauss, M. W., Orsmond, G. I., & Vestal, C. (2000). Families of adolescents and adults with autism: Uncharted territory. International review of research in mental retardation, 23, 267-294.
54. Berthier, M. L., Bayes, A., & Tolosa, E. S. (1993). Magnetic resonance imaging in patients with concurrent Tourette's disorder and Asperger's syndrome. Journal of the American Academy of Child & Adolescent Psychiatry, 32(3), 633-639.
55. Stigler, K. A., & McDougle, C. J. (2013). Structural and functional MRI studies of autism spectrum disorders. The Neuroscience of Autism Spectrum Disorders, 251-266.
56. Allen, G., Müller, R. A., & Courchesne, E. (2004). Cerebellar function in autism: functional magnetic resonance image activation during a simple motor task. Biological psychiatry, 56(4), 269-278.
57. Bartlett, C. W., Gharani, N., Millonig, J. H., & Brzustowicz, L. M. (2005). Three autism candidate genes: a synthesis of human genetic analysis with other disciplines. International Journal of developmental neuroscience, 23(2), 221-234.
58. Yirmiya, N., & Sigman, M. (1991). High functioning individuals with autism: Diagnosis, empirical findings, and theoretical issues. Clinical Psychology Review, 11(6), 669-683.
59. Yu, C. E., Dawson, G., Munson, J., D’Souza, I., Osterling, J., Estes, A., ... & Spence, M. A. (2002). Presence of large deletions in kindreds with autism. The American Journal of Human Genetics, 71(1), 100-115.
60. Murad, A., Fritsch, A., Bizet, É., & Schaal, C. (2014, September). L’autisme à l’âge adulte: bilan diagnostique et aspects thérapeutiques. In Annales Médico-psychologiques, revue psychiatrique (Vol. 172, No. 7, pp. 587-594). Elsevier Masson.
61. Minshew, N. J., & Payton, J. B. (1988). New perspectives in autism. Part 2: The differential diagnosis and neurobiology of autism. Current problems in pediatrics, 18(11), 618-694.
62. Benayed, R., Gharani, N., Rossman, I., Mancuso, V., Lazar, G., Kamdar, S., ... & DiCicco-Bloom, E. (2005). Support for the homeobox transcription factor gene ENGRAILED 2 as an autism spectrum disorder susceptibility locus. The American Journal of Human Genetics, 77(5), 851-868.
63. MacLean, J. E., Szatmari, P., Jones, M. B., Bryson, S. E., Mahoney, W. J., Bartolucci, G., & Tuff, L. (1999). Familial factors influence level of functioning in pervasive developmental disorder. Journal of the American Academy of Child & Adolescent Psychiatry, 38(6), 746-753.
64. Allely, C. S., Hadjikhani, N., Toro, R., & Gillberg, C. (2015). Neuropsychiatry. Brain Mapping, 3, 1049-1060.
65. Suzuki, M., Tachimori, H., Saito, M., Koyama, T., & Kurita, H. (2011). Development of a screening scale for high-functioning pervasive developmental disorders using the Tokyo Child Development Schedule and Tokyo Autistic Behavior Scale. Research in Autism Spectrum Disorders, 5(2), 843-854.
66. Yang, P., Lung, F. W., Jong, Y. J., Hsu, H. Y., & Chen, C. C. (2010). Stability and change of cognitive attributes in children with uneven/delayed cognitive development from preschool through childhood. Research in developmental disabilities, 31(4), 895-902.
67. Nebel-Schwalm, M. S., & Matson, J. L. (2008). Differential diagnosis. Clinical Assessment and Intervention for Autism Spectrum Disorders, 91-129.
68. Szatmari, P., Jones, M. B., Tuff, L., Bartolucci, G., Fisman, S., & Mahoney, W. (1993). Lack of cognitive impairment in first-degree relatives of children with pervasive developmental disorders. Journal of the American Academy of Child & Adolescent Psychiatry, 32(6), 1264-1273.
69. Pharmacological treatment. Assessment and Treatment of Child Psychopathology and Developmental Disabilities, 1, 109-141.
70. Young, R., & Brewer, N. (2002). Diagnosis of autistic disorder: Problems and new directions. International review of research in mental retardation, 25, 107-134.
71. Lam, K. S., Aman, M. G., & Arnold, L. E. (2006). Neurochemical correlates of autistic disorder: a review of the literature. Research in developmental disabilities, 27(3), 254-289.
72. Chapter 3: Assessment. Assessment and Treatment of Child Psychopathology and Developmental Disabilities, 1, 43-58.
73. Swiezy, N., Stuart, M., & Korzekwa, P. (2008). Assessment of independent living / adaptive skills. Clinical Assessment and Intervention For Autism Spectrum Disorders, 193-219.
74. Suzuki, M. (2011). Mental development and autistic behavior in children with pervasive developmental disorders. Research in Autism Spectrum Disorders, 5(4), 1517-1525.
75. Sigafoos, J., Schlosser, R. W., Green, V. A., O’Reilly, M., Lancioni, G. E. (2008). Communication and social skills assessment. Clinical Assessment and Intervention For Autism Spectrum Disorders, 193-219.
76. Shao, Y., Cuccaro, M. L., Hauser, E. R., Raiford, K. L., Menold, M. M., Wolpert, C. M., ... & Abramson, R. K. (2003). Fine mapping of autistic disorder to chromosome 15q11-q13 by use of phenotypic subtypes. The American Journal of Human Genetics, 72(3), 539-548.
77. Etiology and Prevalence. Assessment and Treatment of Child Psychopathology and Developmental Disabilities, 1, 23-41.
78. Oswald, D. P. (1998). Pervasive developmental disorders. Comprehensive Clinical Psychology, 9, 19-35.
79. Piven, J., Chase, G. A., Landa, R., Wzorek, M., Gayle, J., Cloud, D., & Folstein, S. (1991). Psychiatric disorders in the parents of autistic individuals. Journal of the American Academy of Child & Adolescent Psychiatry, 30(3), 471-478.
80. Hooper, S. R., Boyd, T. A., Hynd, G. W., & Rubin, J. (1993). Definitional issues and neurobiological foundations of selected severe neurodevelopmental disorders. Archives of Clinical Neuropsychology, 8(4), 279-307.
81. Index. Journal of the American Academy of Child and Adolescent Psychiatry, 32(6), 1318-1331.
82. Index to Volume 30, 1991. Journal of the American Academy of Child and Adolescent Psychiatry, 30(6), 1034-1043.
83. Campbell, M., Schopler, E., Cueva, J. E., & Hallin, A. (1996). Treatment of autistic disorder. Journal of the American Academy of Child and Adolescent Psychiatry, 35(2), 134-143.
84. Marcelli, D., & Cohen, D. (2016). Autisme infantile et troubles du spectre autistique. Engance et Psychopathologie, 341-394.
85. Subject Index. Comprehensive Psychiatry, 35(6), 480-489.
86. Hagerman, R. J. (1987). Fragile X Syndrome. Current Problems in Pediatrics, 17(11), 626-674.
87. Wong, V. (2002). Abstracts of satellite symposium of the Joint Congress of ICNA & AOCNA 2002 Hong Kong. Brain and Development, 24(5), 315-338.

**Supplementary Materials 3: Targeted papers (Step 2 of Meta-Analysis)**

We identified the following 16 studies as relevant to our meta-analysis. Individual publications are grouped here by the research-group they belong to.

1. *The Ritvo/Bejerot group, Yale University / Karolinska Institutet, Stockholm*

We contacted R. Ritvo, E. Ritvo, and S. Bejerot of the below publications:

Andersen, L. M., Näswall, K., Manouilenko, I., Nylander, L., Edgar, J., Ritvo, R. A., ... & Bejerot, S. (2011). The Swedish version of the Ritvo autism and Asperger diagnostic scale: Revised (RAADS-R). A validation study of a rating scale for adults. Journal of autism and developmental disorders, 41(12), 1635-1645.

Ritvo, R. A., Ritvo, E. R., Guthrie, D., Ritvo, M. J., Hufnagel, D. H., McMahon, W., ... & Eloff, J. (2011). The Ritvo Autism Asperger Diagnostic Scale-Revised (RAADS-R): a scale to assist the diagnosis of Autism Spectrum Disorder in adults: an international validation study. Journal of autism and developmental disorders, 41(8), 1076-1089.

Manouilenko, I., Pagani, M., Stone-Elander, S., Odh, R., Brolin, F., Hatherly, R., ... & Bejerot, S. (2013). Autistic traits, ADHD symptoms, neurological soft signs and regional cerebral blood flow in adults with autism spectrum disorders. Research in Autism Spectrum Disorders, 7(5), 566-578.

1. *The Libero/Kana group, MIND Institute of Sacramento, California / University of Alabama at Birmingham*

We contacted L. Libero and R. Kana of the below publications:

Libero, L. E., Maximo, J. O., Deshpande, H. D., Klinger, L. G., Klinger, M. R., & Kana, R. K. (2014). The role of mirroring and mentalizing networks in mediating action intentions in autism. Molecular autism, 5(1), 50.

Libero, L. E., DeRamus, T. P., Lahti, A. C., Deshpande, G., & Kana, R. K. (2015). Multimodal neuroimaging based classification of autism spectrum disorder using anatomical, neurochemical, and white matter correlates. Cortex, 66, 46-59.

Libero, L. E., Reid, M. A., White, D. M., Salibi, N., Lahti, A. C., & Kana, R. K. (2015). Biochemistry of the cingulate cortex in autism: An MR spectroscopy study. Autism Research.

Libero, L. E., Burge, W. K., Deshpande, H. D., Pestilli, F., & Kana, R. K. (2016). White Matter Diffusion of Major Fiber Tracts Implicated in Autism Spectrum Disorder. Brain Connectivity, 6(9), 691-699.

1. *The Kirkovski/Fitzgerald group, Monash University, Australia / Deacon University, Australia*

We contacted M. Kirkovski of the below publications:

Kirkovski, M., Enticott, P. G., Maller, J. J., Rossell, S. L., & Fitzgerald, P. B. (2015). Diffusion tensor imaging reveals no white matter impairments among adults with autism spectrum disorder. Psychiatry Research: Neuroimaging, 233(1), 64-72.

Kirkovski, M., Enticott, P. G., Hughes, M. E., Rossell, S. L., & Fitzgerald, P. B. (2016). Atypical neural activity in males but not females with autism spectrum disorder. Journal of autism and developmental disorders, 46(3), 954-963.

Kirkovski, M., Rogasch, N. C., Saeki, T., Fitzgibbon, B. M., Enticott, P. G., & Fitzgerald, P. B. (2016). Single pulse transcranial magnetic stimulation-electroencephalogram reveals no electrophysiological abnormality in adults with high-functioning autism spectrum disorder. Journal of child and adolescent psychopharmacology, 26(7), 606-616.

1. *The Schwartzman / Kapp group, University of California, Los Angeles, USA*

We contacted B. Schwartzman of the below publication:

Schwartzman, B. C., Wood, J. J., & Kapp, S. K. (2016). Can the Five Factor Model of Personality Account for the Variability of Autism Symptom Expression? Multivariate Approaches to Behavioral Phenotyping in Adult Autism Spectrum Disorder. Journal of autism and developmental disorders, 46(1), 253-272.

1. *The Schneider/Dux group, University of Queensland, Australia*

We contacted D. Schneider and P. Dux of the below publication:

Schneider, D., Slaughter, V. P., Bayliss, A. P., & Dux, P. E. (2013). A temporally sustained implicit theory of mind deficit in autism spectrum disorders. Cognition, 129(2), 410-417.

1. *The Sizoo/Horwitz group, Dimence, The Netherlands / University Medical Centre Groningen, The Netherlands*

We contacted B. Sizoo and Dr Horwitz of the below publications:

Sizoo, B. B., Horwitz, E. H., Teunisse, J. P., Kan, C. C., Vissers, C. T. W. M., Forceville, E. J. M., ... & Geurts, H. M. (2015). Predictive validity of self-report questionnaires in the assessment of autism spectrum disorders in adults. Autism, 19(7), 842-849.

Sizoo, B., & Horwitz, E. (2014). EPA-0548-Assessment of the psychometric properties of the raads-r and two abridged versions of the autism spectrum quotient (AQ-10 and AQ-28) in a dutch adult outpatient population. European Psychiatry, 29, 1.

1. *The Zimmerman/Gullo group, Griffith University, Australia / University of Queensland, Australia*

We contacted D. Zimmerman and Dr Gullo of the below publication:

Zimmerman, D. L., Ownsworth, T., O'Donovan, A., Roberts, J., & Gullo, M. J. (2016). Independence of hot and cold executive function deficits in high-functioning adults with autism spectrum disorder. Frontiers in human neuroscience, 10.

1. *The Dunlop/Rajan group, Monash University, Australia*

We contacted R. Rajan of the below publication:

Dunlop, W. A., Enticott, P. G., & Rajan, R. (2016). Speech Discrimination Difficulties in High-Functioning Autism Spectrum Disorder Are Likely Independent of Auditory Hypersensitivity. Frontiers in Human Neuroscience, 10.

**Supplementary Materials 4: Invitation to participate in meta-analysis**

*Dear Dr*

*Please forgive me contacting you out of the blue. I am a researcher based at Bournemouth University, UK, studying sex differences in autism. Specifically, I hope to complete a meta-analysis of sex differences as they emerge in the Ritvo Adult Asperger Diagnostic Scale Revised (Ritvo et al, 2011), and thus am contacting authors whom have used this instrument in the hope that they might share part of their data.*

*Your below publication came to my attention during my literature search:*

*…*

*I wondered if you might be willing to share part of the data from that paper, or any other relevant publication since, for the purpose of my meta-analysis. The data I am specifically looking for is:*

*-       Age, total RAADS-R and RAADS-R domain scores from people with confirmed ASD*

*-       Age, total RAADS-R and RAADS-R domain scores from controls, people without ASD*

*If possible, IQ scores of participants would be wonderful to have, too.*

*I would be terribly grateful if you might respond to my enquiry and let me know if this is data you might be willing to share with me, if the terms of your ethics approval allows. I note that I previously contacted Drs Riva and Edward Ritvo who were unable to give me any data themselves, but encouraged and expressed their interest in my work. If you have any questions about my proposed work, I would be delighted to tell you more.*

*Thank you very much for your time in reading this email.*

*Yours sincerely,*

*Rachel Moseley*
